# Adverse drugs reactions to paracetamol and ibuprofen in children: a 5-year report from a pediatric poison control center in Italy

**DOI:** 10.1186/s13052-023-01427-6

**Published:** 2023-02-14

**Authors:** Marco Marano, Marco Roversi, Flavia Severini, Claudia Memoli, Antonio Musolino, Mara Pisani, Corrado Cecchetti, Alberto Villani

**Affiliations:** 1 Pediatric Poison Control Centre, Bambino Gesù Children’s Hospital, IRCCS, Rome, Italy; 2 Pediatric Intensive Care Unit, Bambino Gesù Children’s Hospital, IRCCS, Rome, Italy; 3 Residency School of Pediatrics, University of Rome Tor Vergata, Rome, Italy; 4 Department of Emergency, Acceptance and General Pediatrics, Bambino Gesù Children’s Hospital, Rome, Italy

**Keywords:** Pediatric poisoning, Antipyretics, Analgesics, Non-steroidal anti-inflammatory drugs, Paracetamol, Acetaminophen, Ibuprofen

## Abstract

**Background:**

This study aimed to analyze all the patients who contacted the hospital’s pediatric poison control center (PPCC) for exposure to ibuprofen and acetaminophen, in order to assess the incidence of any adverse reactions.

**Methods:**

We retrospectively reported the clinical data of children who accessed the PPCC of the Bambino Gesù Children’s Hospital, IRCCS, Rome, from January 1, 2018 to September 30, 2022 due to wrong, accidental or intentional intake of inappropriate doses of acetaminophen and/or ibuprofen. In addition, we compared patients according to the intake of one of the two drugs and reported the trimestral distribution of cases during the study period.

**Results:**

A total of 351 patients accessed the PPCC during the study period. The median age was 3.0 years. Most patients were females (57.8%). The most common reason for inappropriate oral intake of paracetamol or ibuprofen was a wrong use or an accidental intake (78.6%), with a fifth of patients taking the drug with suicidal intent (21.1%). According to the PPCC evaluation, most patients were not intoxicated (70.4%). Hospitalization was required for 30.5% of patients. Adverse reactions were reported in 10.5% of cases, with a similar incidence in patients who took paracetamol or ibuprofen. Nausea and vomiting were the most commonly reported adverse reactions. A higher frequency of moderate intoxication was found in patients who took paracetamol compared to ibuprofen (*p* = 0.001). The likelihood of intoxication was also higher in the paracetamol cohort. A spike of cases was registered at the end of 2021.

**Conclusions:**

We analyze exposures to the two most commonly used pediatric molecules, paracetamol and ibuprofen, to assess the frequency of adverse reactions. We demonstrated that these relatively “safe” drugs may be associated with intoxications and adverse reactions when inappropriately administered.

## Background

Pediatric drug exposures are a frequent cause for evaluation by a poison control center or emergency department (ED). Exposures often occur with drugs in the home, such as antipyretics, analgesics and nonsteroidal anti-inflammatory drugs, which are the third leading cause of exposure in children [1]. The severe acute respiratory syndrome coronavirus 2 (SARS-CoV-2) pandemic in 2020, associated with symptoms such as fever, cough, body aches and pains, headache, and sore throat, has also increased the use of anti-inflammatory drugs. In Italy, these consultations mostly take place through a telephone line, and this is useful to avoid the inappropriate flow into hospital emergency rooms. In other cases, children go directly to the pediatric ED. In the pediatric developmental period, the type of poisoning changes from accidental to intentional, but usually drug exposures are of three types: misuse by caregivers, accidental intake, and intentional action [2, 3]. The most commonly used anti-inflammatory drugs are ibuprofen and paracetamol, whose safety profile makes their use widespread even in pediatric settings. Recent reviews examined the effectiveness of these drugs in fever, respiratory tract inflammation, and asthma, and concluded that a similar are rare when used for short periods (less than 7 days) [4, 5]. Moreover, a meta-analysis including 19 studies found no significant difference between the two agents in the incidence of adverse events in pediatric patients [6]. However, both paracetamol and ibuprofen have known and potentially severe dose-proportional side effects, especially in case of inappropriate use. Paracetamol is metabolized by glucuronidation and sulfation in the liver. However, about 5–10% of the drug is metabolized by cytochrome P450 (CYP450) to a toxic metabolite, N-acetyl-p-benzoquinoneimine, which is responsible for hepatocellular damage; in fact, hepatotoxicity appears to be the most serious of the adverse events associated with paracetamol use in children, but adverse events such as urticaria or maculopapular eruptions have been observed [7]. Ibuprofen, a nonselective cyclooxygenase inhibitor, is the antipyretic currently recommended for use in children along with paracetamol because of its tolerability/efficacy profile. Ibuprofen is better tolerated than other nonsteroidal drugs, although it has been associated with the development of renal toxicity, allergic reactions, and gastrointestinal adverse effects. It has also been documented in the literature that ibuprofen use could lead to exacerbation of symptoms in febrile children with a previous history of asthma [8]. The Italian Drug Agency (AIFA) Group reported an increase in suspected adverse reactions possibly related to the use of ibuprofen in parallel with the significant increase in consumption, as it is an over-the-counter drug [9]. In this study we researched and listed all adverse reactions that occurred in a pediatric population, over a 5-year period, and managed by the PPCC of the Bambino Gesù Children’s Hospital in Rome.

## Methods

We retrospectively reported the clinical data of children who accessed the PPCC of the Bambino Gesù Children’s Hospital, IRCCS, Rome, from January 1, 2018 to September 30, 2022 due to wrong, accidental or intentional intake of inappropriate doses of paracetamol and/or ibuprofen. The Poison Control Centre of the Bambino Gesù Children’s Hospital is a national reference point for anyone needing counselling on paediatric poisoning, either through a telephone line that can be accessed by parents and general practitioners/hospital doctors, or through direct access to the hospital’s pediatric emergency room. The doctors involved in the management of intoxicated children are specialists in pediatrics, pediatric intensive care, and clinical pharmaco-toxicology. This multidisciplinary approach allows rapid and effective intervention in the severely intoxicated child with advanced treatments, such as mechanical ventilation, gastroduodenoscopy, dialysis, and extracorporeal circulation.

The following demographic and clinical data were collected from each patient’s medical records: age (years); sex; type of contact (phone call, admission to pediatric Emergency Department or both); reason for intake (wrong use by parents or caregiver, accidental intake, suicidal intent); drug taken (paracetamol, ibuprofen or both); number of patients who took more than 1 molecule (apart from paracetamol/ibuprofen); number of symptomatic patients; type of drug intake (ingestion, mucosal contact, injection, other); place of intake (at home, at hospital, other/unknown); likelihood of intoxication; grading of intoxication; hospitalization (brief intensive observation area, standard pediatric department, pediatric intensive care unit); adverse reactions (related to paracetamol or ibuprofen intake, reported and not reported in drug data sheet). We compared patients according to intake of paracetamol and ibuprofen and according to suicidal or non-suicidal intent of intake. The distribution of cases in trimesters during the study period was also reported to show the association with specific time intervals or annual recurrences.

The likelihood of intoxication was defined according to the Toxic Exposure Surveillance System developed by the CDC [10].

- *not intoxicated*, if the relationship between effects and exposure is not compatible with the known toxicological characteristics of the agent;
- *suspected intoxication*, if there is objective or subjective evidence of effects and/or exposure, whereby the toxicological information about the agent is insufficient to determine a causal relationship causal relationship between the observed manifestations and the agent;
- *possible intoxication*, if only subjective evidence is available on both effects and exposure, and the effects detected are compatible with both the exposure times and the the known toxicological characteristics of the agent;
- *probable intoxication*, if objective evidence is available on the effects but only subjective data on the occurrence of absorption are available;
- *certain intoxication*, if the available objective evidence can confirm both the effects and the occurrence of exposure, and the effects detected are compatible with both the timing of exposure and with the toxicological characteristics of the agent;
- *not indicated*, if the available documentation on effects and exposure is insufficient to determine a possible association.

The severity of intoxication is assessed by following the guidance provided by the CDC [10], defined readjusting the criteria published by Persson et al. [11] and adopted by the International Programme on Chemical Safety. Cases of certain, probable, possible is suspected intoxication are classified into the following categories:

- *mild*, for cases that usually manifest skin, eye or respiratory tract irritation upper, but may also manifest fever, headache, fatigue, dizziness, all regressing without treatment;
- *moderate*, for cases with less severe effects, often with systemic manifestations, that generally require therapy;
- *severe*, for cases with effects severe enough to jeopardize survival of patients or cause permanent injury, including, but not limited to, coma, cardiac arrest, renal failure, and/or respiratory depression.

### Statistical analysis

Statistical analysis was performed with IBM SPSS Statistics software version 23.0 (IBM Corp. Armonk, NY). A two-tailed *p*-value < 0.05 was considered for statistical significance. All continuous variables were summarized with medians, interquartile ranges (IQRs) and 25th–75th percentile intervals and compared with the Mann-Whitney U-test. All categorical variables were expressed as absolute numbers and relative percentages and compared by chi-square test and Fisher’s exact test, as appropriate.

## Results

A total of 351 patients accessed the PCC during the study period. In the overall cohort, 37 adverse reactions were recorded (10.5%), occurring equally in the paracetamol and ibuprofen cohorts. The demographic and clinical characteristics of the patients are outlined in Table 1.

**Table 1:** Characteristics of study sample

Total – no. (%)	351
Age (years) - median (IQR, 25° - 75°)	3.0 (8.1, 1.7–9.8)
Sex (females)	203 (57.8)
Male to female ratio	1:1.4
Type of contact	
- Phone call	223 (63.5)
- Admission to ED	121 (34.5)
- Both	7 (2.0)
Reason for intake	
- Wrong use	144 (41.0)
- Accidental intake	132 (37.6)
- Suicidal intent	74 (21.1)
- Correct use^a^	1 (0.3)
Drug taken	
- Paracetamol	230 (65.5)
- Ibuprofene	119 (33.9)
- Both	2 (0.6)
More than 1 molecule taken^b^	21 (6.0)
Symptomatic	26 (7.4)
Comorbidities	69 (19.7)
Type of drug intake	
- Ingestion	309 (88.0)
- Oral/mucosal	10 (2.8)
- Injection	1 (0.3)
- Other	31 (8.8)
Place of intake	
- At home	342 (97.4)
- At hospital	2 (0.6)
- Other/unknown	7 (2.0)
Grading of intoxication	
- Mild	275 (78.3)
- Moderate	66 (18.8)
- Severe	8 (2.3)
- NA	2 (0.5)
Likelihood of intoxication	
- Not intoxicated	247 (70.4)
- Suspected intoxication	40 (11.4)
- Possible intoxication	25 (7.1)
- Probable intoxication	15 (4.3)
- Certain intoxication	13 (3.7)
- Not indicated	11 (3.1)
Hospitalization	107 (30.5)
- Pediatric department	62 (57.9)
- Brief intensive observation	43 (40.2)
- Pediatric intensive care unit	1 (0.9)

^a^Agitation in a child after administration of a standard dose of paracetamol

^b^Apart from paracetamol or ibuprofen

The median age was 3.0 years (IQR 3.0). Most patients were females (57.8%). The most frequent type of contact with the PPCC was by phone (63.5%). A third of all patients (34.5%) physically accessed the PPCC. The most common reason for inappropriate intake of paracetamol or ibuprofen was a wrong use (41.0%) or an accidental intake (37.6%). Approximately a fifth of patients took the drug with suicidal intent (21.1%). Most children took paracetamol (65.5%), mainly by ingestion (88.0) and at home (97.4%). According to the PPCC evaluation, most patients were not intoxicated (70.4%), with only a minority being attributed a probable or certain intoxication (8.0%). Hospitalization was required for 107 patients (30.5%): 62 were admitted to a standard pediatric department and 43 only required brief intensive observation.

The reported adverse reactions are described in Table 2.

**Table 2:** Adverse reactions by drug

Total – no. (%)	37 (10.5)
Paracetamol	25/232 (10.8)^a^
Reported in drug data sheet	
- Nausea	11 (44)
- Headache	3 (12)
- Agitation	2 (8)
- Liver failure	1 (4)
Not reported in drug data sheet	
- Hypertransaminasemia	6 (24)
- Vomiting	6 (24)
- Coagulopathy	3 (12)
- Abdominal pain	2 (8)
- Increased creatinine	1 (4)
- QT elongation	1 (4)
- Sleepiness	1 (4)
Ibuprofen	13/119 (10.1)*
Reported in drug data sheet	
- Dizziness	3 (23)
- Nausea	3 (23)
- Acute renal failure	1 (8)
- Thrombocytopenia	1 (8)
Not reported in drug data sheet	
- Abdominal pain	4 (31)
- Vomiting	4 (31)
- Increased creatinine	2 (15)
- Agitation	1 (8)
- Albuminuria	1 (8)
- Coagulopathy	1 (8)
- Hypertransaminasemia	1 (8)
- Hypotension	1 (8)
- Increased CPK	1 (8)

*Two patients took both paracetamol and ibuprofen and one developed some side effects that could not be attributed to either drug and were therefore counted for each

Adverse reactions were reported in 37 cases (10.5%), with a similar incidence in patients who took paracetamol (10.9%) and those who took ibuprofen (10.1%). Nausea was the most commonly reported adverse reaction in the paracetamol cohort (44%), followed by hypertransaminasemia (24%), vomiting (24%), headache (12%), and coagulopathy (12%). In patients who took ibuprofen, abdominal pain (31%), vomiting (31%), dizziness (23%), and nausea (23%) were the most commonly reported adverse reactions.

The comparison between patients who took paracetamol and ibuprofen is reported in Table 3.

**Table 3:** Comparison of patients who took paracetamol and ibuprofen^a^

	Paracetamol	Ibuprofen	***p***-value
**Total – no. (%)**	**230**	**119**	
Age (years) - median (IQR, 25° - 75°)	2.9 (12.1, 1.3–13.3)	3.2 (5.1, 2.2–7.3)	0.191
Sex (females)	138 (60.0)	63 (52.9)	0.206
Male to female ratio	1:1.5	1:1.1	
Type of contact			
- Phone call	139 (60.4)	84 (70.6)	0.061
- Admission to ED	86 (37.4)	33 (27.7)	0.071
- Both	5 (2.2)	2 (1.7)	1.000
Reason for intake			
- Wrong use	105 (45.7)	39 (32.8)	**0.021**
- Accidental intake	67 (29.1)	65 (54.6)	**< 0.001**
- Suicidal intent	57 (24.8)	15 (12.6)	**0.008**
- Correct use*	1 (0.4)	–	1.000
More than 1 molecule taken**	14 (6.1)	5 (4.2)	0.462
Symptomatic	19 (8.3)	7 (5.9)	0.422
Comorbidities	51 (22.2)	18 (15.1)	0.117
Type of drug intake			
- Ingestion	189 (82.2)	118 (99.2)	**< 0.001**
- Oral/mucosal	10 (4.3)	–	**0.018**
- Injection	1 (0.4)	–	1.000
- Other	30 (13.0)	1 (0.8)	**< 0.001**
Place of intake			
- At home	226 (98.3)	115 (96.6)	0.452
- At hospital	2 (0.9)	–	0.549
- Other/unknown	2 (0.9)	4 (3.4)	0.186
Grading of intoxication			
- Mild	169 (73.5)	106 (89.1)	**0.001**
- Moderate	53 (23.0)	11 (9.2)	**0.001**
- Severe	7 (3.0)	1 (0.8)	0.273
- NA	1 (0.4)	1 (0.8)	1.000
Likelihood of intoxication			
- Not intoxicated	155 (67.4)	92 (77.3)	0.053
- Suspected intoxication	24 (10.4)	14 (11.8)	0.705
- Possible intoxication	22 (9.6)	3 (2.5)	**0.016**
- Probable intoxication	11 (4.8)	3 (3.4)	0.535
- Certain intoxication	13 (5.7)	–	**0.006**
- Not indicated	5 (2.2)	6 (5.0)	0.195
Hospitalization			
- Pediatric department	47 (60.3)	14 (51.9)	0.446
- Brief intensive observation	30 (38.5)	13 (48.1)	0.378
- Pediatric intensive care unit	1 (1.3)	–	1.000

^a^Two cases of both paracetamol and ibuprofen intake were removed

A wrong use of paracetamol was more frequent than ibuprofen (*p* = 0.021), whose intake was more often accidental (*p* < 0.001). A suicidal intent was more common with the use of paracetamol (*p* = 0.008). Correspondingly, a higher frequency of moderate intoxication was found in patients who took paracetamol compared to ibuprofen (*p* = 0.001). The likelihood of intoxication was also higher in the paracetamol cohort (*p* = 0.016 for “possible” and *p* = 0.006 for “certain” intoxication).

The comparison between patients who took any drug with and without a suicidal intent is reported in Table 4.

**Table 4:** Comparison of patients with suicidal and non-suicidal intent*

	Suicidal intent	Non-suicidal intent	***p***-value
**Total – no. (%)**	**74**	**277**	
During/after COVID-19 pandemic	50 (67.6)	112 (40.4)	**< 0.001**
Age (years) - median (IQR, 25° - 75°)	15.8 (1.9, 14.6–16.5)	2.5 (2.7, 1.3–3.9)	**< 0.001**
Sex (females)	70 (94.6)	133 (48.0)	**< 0.001**
Male to female ratio	1:17.5	1:0.9	
Type of contact			
- Phone call	3 (4.1)	220 (79.4)	**< 0.001**
- Admission to ED	69 (93.2)	52 (18.8)	**< 0.001**
- Both	2 (2.7)	5 (1.8)	0.641
Paracetamol	59 (79.7)	173 (62.5)	**0.005**
Ibuprofen	17 (23.0)	104 (37.5)	**0.019**
More than 1 molecule taken**	18 (24.3)	3 (1.1)	**< 0.001**
Symptomatic	20 (27.4)	7 (2.5)	**< 0.001**
Comorbidities	49 (66.2)	20 (7.2)	**< 0.001**
Type of drug intake			
- Ingestion	74 (100)	235 (84.8)	**< 0.001**
- Oral/mucosal	–	10 (3.6)	0.129
- Injection	–	1 (0.4)	1.000
- Other	–	31 (11.2)	**0.003**
Place of intake			
- At home	72 (97.3)	271 (97.8)	0.677
- At hospital	–	2 (0.7)	1.000
- Other/unknown	2 (2.7)	4 (1.4)	0.610
Adverse reactions	27 (36.5)	9 (3.2)	**< 0.001**
Grading of intoxication			
- Mild	17 (23.0)	258 (93.1)	**< 0.001**
- Moderate	50 (67.6)	16 (5.8)	**< 0.001**
- Severe	7 (9.5)	1 (0.4)	**< 0.001**
- NA	–	2 (0.7)	1.000
Likelihood of intoxication			
- Not intoxicated	13 (17.6)	234 (84.5)	**< 0.001**
- Suspected intoxication	22 (29.7)	18 (6.5)	**< 0.001**
- Possible intoxication	16 (21.6)	9 (3.2)	**< 0.001**
- Probable intoxication	12 (16.2)	3 (1.1)	**< 0.001**
- Certain intoxication	10 (13.5)	3 (1.1)	**< 0.001**
- Not indicated	1 (1.4)	10 (3.6)	0.470
Hospitalization			
- Pediatric department	55 (77.5)	8 (22.2)	**< 0.001**
- Brief intensive observation	15 (21.1)	28 (77.8)	**< 0.001**
- Pediatric intensive care unit	1 (1.4)	–	1.000

More patients with suicidal intent were admitted during or after the COVID-19 pandemic (adopting 9 March 2020 as the Italian target date for the start of the pandemic) than patients with non-suicidal intent (67.6 vs 40.4%, *p* < 0.001). Patients with suicidal intent were also significantly older (median age 15.8 vs 2.5 years, *p* < 0.001) and more frequently females (94.6 vs 48.0%, *p* < 0.001). Paracetamol (79.7 vs 62.5%, *p* = 0.005) was more frequently associated with suicidal intent than ibuprofen (23.0 vs 37.5%, *p* = 0.019). Not surprisingly, suicidal intent was more frequently associated with intake of more than one molecule (24.3 vs 2.5%, *p* < 0.001) and with comorbidities (66.2 vs 7.2%, < 0.001). Moreover, they were subjected to adverse reactions significantly more often than children with non-suicidal intent (36.5 vs 3.2%, *p* < 0.001). A higher grade and likelihood of intoxication was found in patients with suicidal intent (see Table 4 for more details). Most patients who took either paracetamol or ibuprofen with suicidal intent were admitted to a pediatric department (77.5 vs 22.2%, < 0.001); only a minority was briefly observed before discharge (21.1 vs 77.8%, *p* < 0.001).

**Fig. 1:**
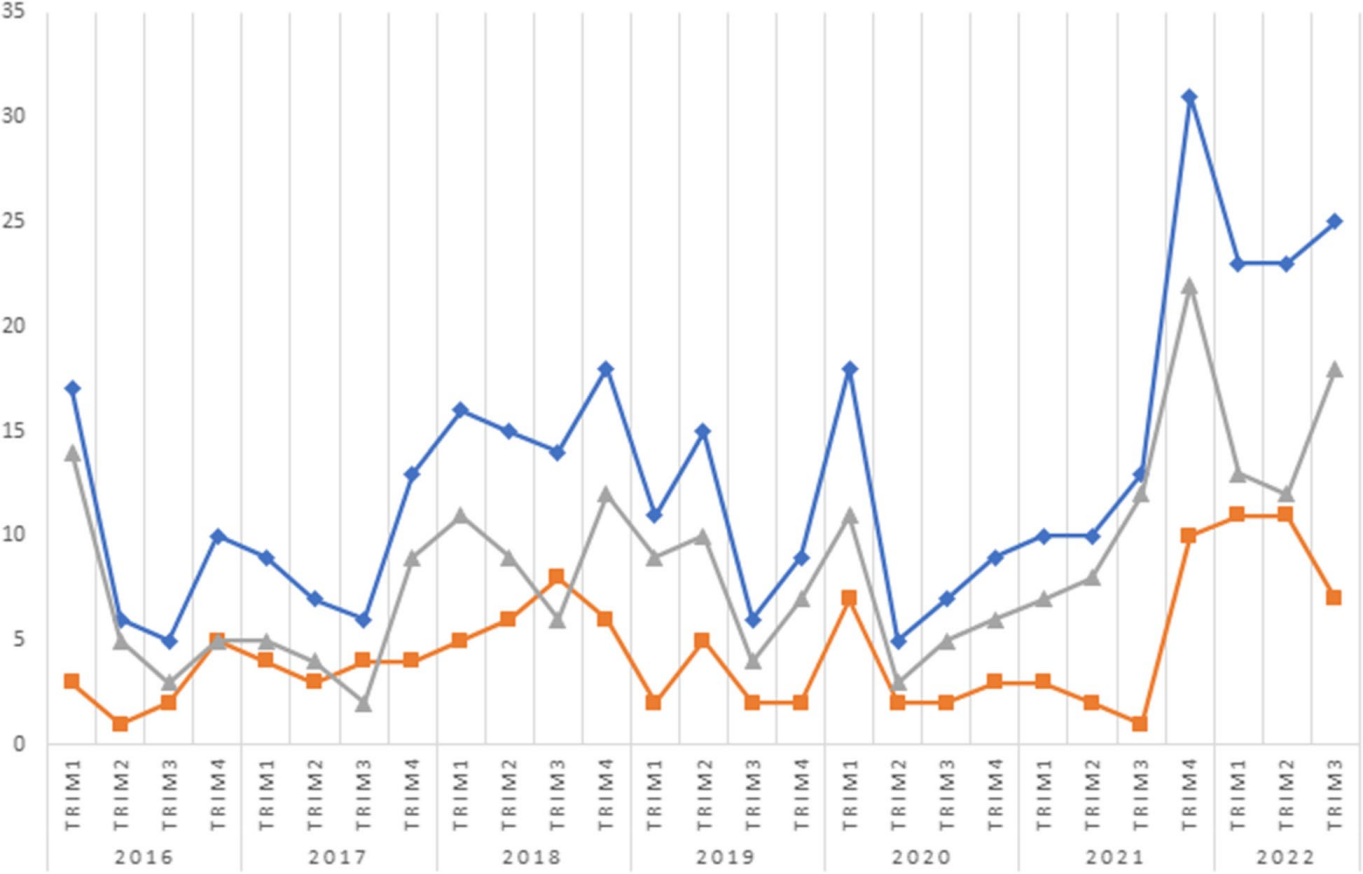
Distribution of cases per trimester: total cases (blue), paracetamol (gray), ibuprofen (orange)

The distribution of cases into quarters, as shown in Fig. 1, demonstrated a steady number of cases over the years, with the exception of the current year and the end of 2021, when a spike of cases was registered.

## Discussion

Our study shows that younger children are at risk of taking an inappropriate dose of paracetamol or ibuprofen, mainly due to wrong or accidental administration by parents at home. These events mostly lead to telephone contact with the PPCC and, in one-third of cases (34.5%), direct referral to the emergency department. However, suicidal intent was traced in one-fifth of cases (21.1%). Only a minority of patients were symptomatic (7.4%), with equal incidences between the paracetamol and ibuprofen cohorts. Correspondingly, most patients were evaluated as mildly (78.3%) or not intoxicated (70.4%). Only less than a third of patients required hospitalization (30.5%). However, 40.2% of these were just observed briefly after ED admission and then discharged.

In the overall cohort, 37 adverse reactions were recorded (10.5%), occurring equally in the paracetamol and ibuprofen cohorts. The most common adverse reactions in children taking either paracetamol or ibuprofen were gastrointestinal in nature: nausea and vomiting in both cases being the most representative symptoms.

Comparison of children who took paracetamol or ibuprofen showed that the former was more frequently associated with wrong use (*p* = 0.021) and suicidal intent (*p* = 0.008) and less frequently with accidental intake (*p* < 0.001). Intake of paracetamol was also more frequently associated with a moderate (*p* = 0.001), rather than a mild (*p* = 0.001) intoxication, compared to ibuprofen. This may be due to the more frequent suicidal intent behind paracetamol use, both due to local popularity and greater availability of this over-the-counter drug at home. More specifically, as most Italian pediatricians are aware of the risk linked to inappropriate use of paracetamol and ibuprofen, correctly indicating the former as first choice against fever [12], the commonly reported wrong use of paracetamol is probably due to an inappropriate autonomous administration. Moreover, the toxic dose of ibuprofen is higher than that of paracetamol, and requires greater availability of the product at home [13]. In particular, an oral dose of 200 mg/kg (or 12 g) of paracetamol is considered toxic [14], while a dose of 400 mg/kg is required for ibuprofen-mediated toxicity [15].

The COVID-19 pandemic caused a surge in neuropsychiatric disorders among children, with a parallel increase in suicide attempts, as demonstrated in our study, consistently with other reports [16, 17]. Our results are self-explanatory: children with suicidal intent were older and more likely to experience drug adverse reactions and to be intoxicated, coherently with a higher intake of paracetamol and ibuprofen. Interestingly, paracetamol assumption was more frequently associated with suicidal intent than ibuprofen, probably owing to the widespread availability of paracetamol and the known severe hepatotoxicity associated with its use.

The distribution of cases in trimesters showed a remarkable increase during the last trimester of 2021 and first trimester of 2022. It is difficult to address this increase, but a possible link to the recent pandemic and, more specifically, to the broad reduction of preventive measures after the successful vaccination campaign against COVID-19 cannot be ruled out. Moreover, the increased availability of these drugs at home, purchased during the pandemic to alleviate the symptoms of COVID-19, may have contributed to their uncontrolled self-administration.

Our article is limited by its retrospective nature and lack of definition of weight-adjusted doses and, therefore, toxicity levels. However, it contributes to the safety profiling of drugs widely used in pediatrics, such as paracetamol and ibuprofen, by providing real-world data.

## Conclusions

Our center is one of the major Italian Poison Control Centers and the only one dedicated exclusively to children. We wanted to analyze exposures to the two most commonly used pediatric molecules, paracetamol and ibuprofen, to assess the frequency of adverse reactions. We demonstrated that these relatively “safe” drugs may be associated with intoxications and adverse reactions when inappropriately and/or autonomously administered. We therefore recommend that paracetamol and ibuprofen be administered only when necessary, following the doses and instructions of the treating pediatrician.

## Data Availability

The datasets used and/or analysed during the current study are available from the corresponding author on reasonable request.

## Abbreviations

CDC: Center for Disease Control and Prevention
ED: Emergency Department
PCC: Poison Control Center
